# Prognostic value of lymph node to primary tumor standardized uptake value ratio in unresectable esophageal cancer

**DOI:** 10.1186/s12885-020-07044-4

**Published:** 2020-06-10

**Authors:** Po-Jui Chen, Wing-Keen Yap, Yu-Chuan Chang, Chen-Kan Tseng, Yin-Kai Chao, Jason Chia-Hsun Hsieh, Ping-Ching Pai, Ching-Hsin Lee, Chan-Keng Yang, Albert Tsung-Ying Ho, Tsung-Min Hung

**Affiliations:** 1 Department of Radiation Oncology and Proton Therapy Center, Linkou Chang Gung Memorial Hospital, 5 Fu-Shin Street, Kwei-Shan, Taoyuan, Taiwan; 2 Department of Nuclear Medicine and Molecular Imaging Center, Linkou Chang Gung Memorial Hospital, Taoyuan, Taiwan; 3 Department of Medical Imaging and Radiological Sciences, College of Medicine, Chang Gung University, No.259, Wenhua 1st Rd., Kwei-Shan, Taoyuan, Taiwan; 4 Division of Thoracic Surgery, Department of Surgery, Linkou Chang Gung Memorial Hospital, Taoyuan, Taiwan; 5 Division of Medical Oncology, Department of Internal Medicine, Linkou Chang Gung Memorial Hospital, Taoyuan, Taiwan; 6 Department of Chemical and Materials Engineering, Chang Gung University, No.259, Wenhua 1st Rd., Kwei-Shan, Taoyuan, Taiwan

**Keywords:** Esophageal cancer, 2-deoxy-2-[18F]fluoro-D-glucose positron emission tomography (FDG PET), Maximum standardized uptake value (SUV), Ratio, Prognosis, Unresectable, Squamous cell carcinoma (SCC), Distant metastasis, Node-to-tumor SUV ratio (NTR), Personalized treatment

## Abstract

**Background:**

Unresectable esophageal cancer harbors high mortality despite chemoradiotherapy. Better patient selection for more personalized management may result in better treatment outcomes. We presume the ratio of maximum standardized uptake value (SUV) of metastatic lymph nodes to primary tumor (NTR) in 2-deoxy-2-[18F]fluoro-D-glucose positron emission tomography/computed tomography (FDG PET/CT) may provide prognostic information and further stratification of these patients.

**Methods:**

The patients with non-metastatic and unresectable esophageal squamous cell carcinoma (SCC) receiving FDG PET/CT staging and treated by chemoradiotherapy were retrospectively reviewed. Receiver operating characteristic (ROC) analysis was performed to determine the optimal cut-off value for NTR. Kaplan-Meier method and Cox regression model were used for survival analyses and multivariable analyses, respectively.

**Results:**

From 2010 to 2016, 96 eligible patients were analyzed. The median follow-up time was 10.2 months (range 1.6 to 83.6 months). Using ROC analysis, the best NTR cut-off value was 0.46 for prediction of distant metastasis. The median distant metastasis-free survival (DMFS) was significantly lower in the high-NTR group (9.5 vs. 22.2 months, *p* = 0.002) and median overall survival (OS) (9.5 vs. 11.6 months, *p* = 0.013) was also significantly worse. Multivariable analysis revealed that NTR was an independent prognostic factor for DMFS (hazard ratio [HR] 1.81, *p* = 0.023) and OS (HR 1.77, *p* = 0.014).

**Conclusions:**

High pretreatment NTR predicts worse treatment outcomes and could be an easy-to-use and helpful prognostic factor to provide more personalized treatment for patients with non-metastatic and unresectable esophageal SCC.

## Background

Globally, there were 572,000 newly diagnosed esophageal cancer cases and an estimated 509,000 deaths, which makes it the sixth leading cause of cancer death in 2018 [1]. Eastern Asia has the highest incidence at 17.9/100,000 people per year and squamous cell carcinoma (SCC) accounts for more than 90% cases [2]. Chemoradiotherapy (CRT) has been utilized for unresectable T4b esophageal cancer, which is defined by aorta, trachea, or vertebrae invasion. This approach remains controversial because unresectable diseases were excluded from most previous prospective trials and dismal treatment outcomes.

Not only locoregional but distal failure is a problem for unresectable esophageal cancer. Around half of esophageal cancer patients had distant metastasis at diagnosis, and one-third of patients would develop distant metastasis after the radical treatment [3]. However, there are no strong pretreatment prognostic factors available for unresectable esophageal cancer. A retrospective study from the National Cancer Database conducted by Cushman et al. [4] revealed that traditional risk factors like age, histology, and clinical nodal stage had no significant impact on overall survival in unresectable esophageal cancer. Yamaguchi et al. [5] reported only clinical N0 and later treatment period were related to a better prognosis. The paucity of reliable prognostic factors for unresectable esophageal cancer made us seek for a more potent prognosticator.

2-deoxy-2-[18F]fluoro-D-glucose positron emission tomography/computed tomography (FDG PET/CT) played an indispensable role in esophageal cancer staging and its primary usefulness is to detect distant metastasis [6]. Utilizing PET/CT to predict the patient’s prognosis has been an attractive idea. Among all the PET parameters, maximum standardized uptake value (SUV) and its derivatives received the most attention for prognosis prediction because of the convenience. MUNICON phase II trial [7] first utilized PET for response evaluation of neoadjuvant chemotherapy for adenocarcinoma of esophagogastric junction. As for SCC, our previous study [8] revealed that PET response after CRT was predictive for distant metastasis-free survival (DMFS) and overall survival (OS) while Greally et al. [9] reported that PET response after induction chemotherapy was predictive for progression-free survival (PFS) and OS. The value of SUV in neoadjuvant treatment response evaluation was well demonstrated in a review of 26 published studies [10].

The ratio of metastatic lymph nodes SUV (SUV_LN_) to primary tumor SUV (SUV_Tumor_), i.e. node-to-tumor SUV ratio (NTR), is a promising functional biomarker which could be easily acquired without much change of the established work-flow and easily accessible for a retrospective study. NTR has been evaluated for prediction of axillary macrometastasis and prognosis in breast cancer [11, 12], nodal staging in non-small-cell lung cancer [13], and prognosis prediction in cervical and endometrial cancer [14, 15]. We presumed that NTR may also provide prognostic information in patients with unresectable esophageal SCC.

## Methods

### Patients

The institutional review board approved this retrospective study, which enrolled patients with unresectable esophageal SCC without distal metastasis (i.e., cT4bN0-3 M0, according to American Joint Committee on Cancer 7th edition) with histopathological confirmation between December 2010 and June 2016 from the database of the radiation oncology department. All clinical data were collected from the electronic medical record. The pretreatment workup included chest and abdominal CT, endoscopy with or without ultrasound, and FDG PET/CT. The primary tumor length was measured with the CT scan and was correlated with, if available, the endoscopy finding. Exclusion criteria included absence of FDG PET/CT at staging, radiotherapy or chemotherapy alone treatment, and recurrent esophageal cancer. Patients with previous cancer history but without evidence of disease for more than two years at the time of esophageal cancer diagnosis were enrolled.

### FDG PET/CT protocols

The patients were asked to fast for at least 4 h before the examination. Depending on subject’s body weight, 200–444 MBq of [^18^F] FDG were injected intravenously. The images were acquired 90 min after the tracer injection. Both PET and low-dose CT covered the skull vertex to the middle thigh. PET was performed on a Biograph mCT scanner (Siemens Medical Solution) or a Discovery ST16 scanner (GE Healthcare), and the images were reconstructed using low-dose CT-based attenuation correction. Each PET scan was acquired with ordered-subset expectation maximization (OSEM) iterative reconstruction algorithm (4 iterations and 10 subsets for the Discovery ST16; 2 iterations and 21 subsets for the Biography mCT). The axial spatial resolutions of PET at the center were 2.16 and 4.80 mm for Biograph mCT and Discovery ST16, respectively. The fusion of low-dose CT and PET images were used for interpretation. The standardized uptake value was calculated according to the following formula: standardized uptake value = radioactivity concentration in tissue [becquerel/gram]/ (injected dose [becquerel]/patient weight [gram]). The maximal standardized uptake value of the primary tumor and metastatic lymph nodes, i.e., SUV_Tumor_ and SUV_LN_, were obtained separately within the volumes-of-interest created manually. The NTR was defined as (SUV_LN_ / SUV_Tumor_).

### Treatment

CRT was given to all patients. The chemotherapy regimens could be cisplatin plus 5-fluorouracil (5-FU), paclitaxel plus carboplatin, or cisplatin plus paclitaxel. The total dose of radiotherapy was targeted to be 41.4–60 Gy in 23–30 fractions but both split-course or conventional continuous radiotherapy were accepted. The decision of chemotherapy and radiotherapy protocols were based on the physician’s preference.

After the conventional continuous or first course of split-course radiotherapy, the patients were encouraged to receive post-treatment evaluation with chest and abdominal CT, endoscopy with or without ultrasound, and FDG PET/CT. For those who have resectable diseases after the CRT, radical esophagectomy was suggested to be performed. For patients with sustained unresectable disease or unwilling to have the operation, if receiving first course of split-course radiotherapy at the start, the second course of CRT would be given to achieve the planned total dose of radiotherapy as mentioned above.

### Surveillance and clinical endpoints

After the treatment, all patients underwent regular follow-up based on our institutional protocol. Clinic appointments were arranged every 3 months during the first 2 years, every 4–6 months during the third and fourth years, and every 6–12 months thereafter. CT scan and endoscopy were performed every 3–6 months or when clinically indicated.

Four clinical endpoints of this study were OS, PFS, local regional failure-free survival (LRFFS), and DMFS, which were measured from pathologic confirmation to death or events.

### Statistical analysis

Receiver operating characteristic (ROC) analysis was performed to determine the optimal cut-off value for NTR. Chi-square tests, Fisher’s exact tests, and Mann-Whitney U tests were done to compare clinical and pathological parameters. Kaplan-Meier method and log-rank tests were utilized for survival analyses and comparison across different variables. Cox proportional hazard model was used for evaluating the independent influences of prognostic variables. Backward elimination method with Wald statistic was used for the model selection. The probability for exclusion from model was 0.10. All *P* values were two-sided and statistical significance was defined as P value less than 0.05. All statistical analyses were performed using SPSS software (IBM Corp. Released 2017. IBM SPSS Statistics for Macintosh, Version 25.0. Armonk, NY: IBM Corp.).

## Results

### Patient characteristics

From 2010 to 2016, there were 149 patients with unresectable esophageal cancer (T4b) registered in the departmental database. After excluding the patient who did not meet our inclusion criteria mentioned above, 96 eligible patients were collected and analyzed. The median follow-up time was 10.2 months (range 1.6 to 83.6 months, interquartile range [IQR] 6.1 to 26.3 months). The median SUV_Tumor_, SUV_LN_, and NTR were 18.3, 9.2 and 0.52, respectively. Detailed clinical and pathological characteristics were shown in Table 1.

**Table 1:** Patient characteristics before and after grouping by NTR

		Cohort (***n*** = 96)	Low-NTR (***n*** = 44)	High-NTR (***n*** = 52)	***p***-value^**b**^
**Gender**	Male	88 (92%)	41 (93%)	47 (90%)	0.723
	Female	8 (8%)	3 (7%)	5 (10%)	
**Age at diagnosis**^**a**^		53.2 [49.0–58.5]	53.5 [48.9–59.6]	52.7 [49.0–58.2]	0.938
**Previous cancer history**	No	92 (96%)	42 (96%)	50 (96%)	1.000
	Yes	4 (4%)	2 (5%)	2 (5%)	
**Performance status**	ECOG 0–1	93 (97%)	41 (93%)	52 (100%)	0.093
	ECOG 2–3	3 (3%)	3 (7%)	0 (0%)	
**Differentiation**	Well to Moderate	68 (77%)	33 (83%)	35 (73%)	0.318
	Poor	20 (23%)	7 (18%)	13 (27%)	
	Missing	8	4	4	
**Tumor length**^**a**^**(cm)**		7.0 [5.7–8.4]	7.2 [5.6–9.0]	7.0 [5.8–8.0]	0.459
**Tumor location**	Cervical	22 (23%)	11 (25%)	11 (21%)	0.716
	Upper third	31 (32%)	12 (27%)	19 (37%)	
	Middle third	40 (42%)	19 (43%)	21 (40%)	
	Lower third	3 (3%)	2 (5%)	1 (2%)	
**Nodal stage (AJCC 7th edition)**	N0-N1	14 (15%)	9 (21%)	5 (10%)	**0.028**
	N2	52 (54%)	27 (61%)	25 (48%)	
	N3	30 (31%)	8 (18%)	22 (42%)	
**First RT dose**^**a**^**(Gy)**	< 40 Gy	45 (47%)	14 (32%)	31 (60%)	**0.007**
	> = 40 Gy	51 (53%)	30 (68%)	21 (40%)	
**Chemotherapy regimen**	Cisplatin + 5-FU	68 (71%)	28 (64%)	40 (77%)	0.064
	Carboplatin + Paclitaxel	24 (25%)	12 (27%)	12 (23%)	
	Cisplatin + Paclitaxel	4 (4%)	4 (9%)	0 (0%)	
**SUV**_**Tumor**_^**a**^		18.3 [14.8–23.2]	18.3 [15.6–24.4]	17.8 [13.3–21.7]	0.107
**SUV**_**LN**_^**a**^		9.2 [4.4–14.3]	4.4 [3.1–6.8]	13.7 [10.8–16.8]	**< 0.001**
**NTR**^**a**^		0.52 [0.24–0.80]	0.22 [0.13–0.31]	0.77 [0.65–0.98]	**< 0.001**

*SUVTumor* maximal SUV of primary tumor, *SUVLN* maximal SUV of metastatic node, *NTR* node-to-tumor SUV ratio, *5-FU* 5-fluorouracil

^a^ Values are presented as median [interquartile range, 25th–75th percentile]

^b^ Comparison of high-NTR and low-NTR groups, p values less than 0.05 indicated statistical significance

### Determination of cut-off value and grouping

**Fig. 1:**
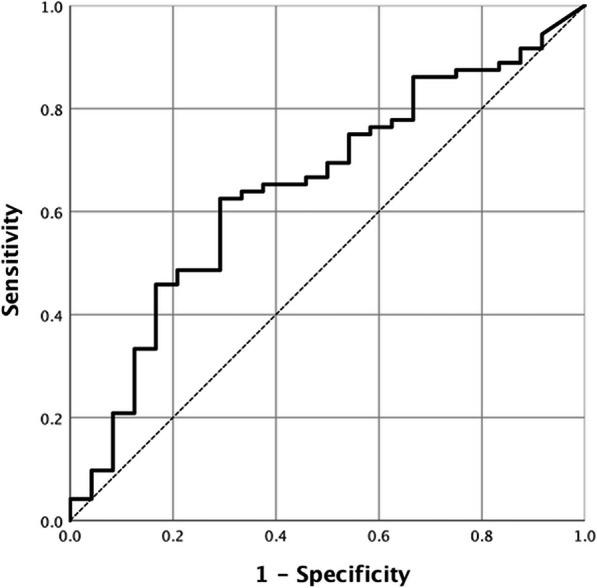
ROC (receiver operating characteristic) curve analysis of distant metastasis-free survival prediction according to the node-to-tumor ratio of SUV (NTR). The area under the curve was 0.648 (95% confidence interval [CI] 0.523–0.773). The best NTR cut-off value was 0.46 for prediction of distant metastasis

Using the ROC analysis and assigning occurrences of distant metastasis as the endpoint, the cut-off value of NTR at 0.46 was acquired and the area-under-curve (AUC) was 0.648 (95% confidence interval [CI] 0.523–0.773, Fig. 1). The sensitivity was 62.5% and the specificity was 70.8%.

After the grouping, there were 44 patients in the low-NTR group and 52 patients in the high-NTR group, respectively. The high-NTR group has significantly more patients with higher nodal stage (*p* = 0.028) and more patients receiving less than 40 Gy in the first course of radiotherapy (32% vs 60%, *p* = 0.007). There were also marginal differences in terms of performance status and chemotherapy regimens. The median SUV_LN_ and NTR were 4.4, 0.22 and 13.7, 0.77 in the low-NTR and high-NTR group, respectively.

In the low-NTR and high-NTR group, there were 10 (22.7%) and 6 (11.5%) patients, respectively, received radical esophagectomy after the CRT. There was no statistical difference in surgery rate between groups (*p* = 0.143).

### Prediction of survival

**Fig. 2:**
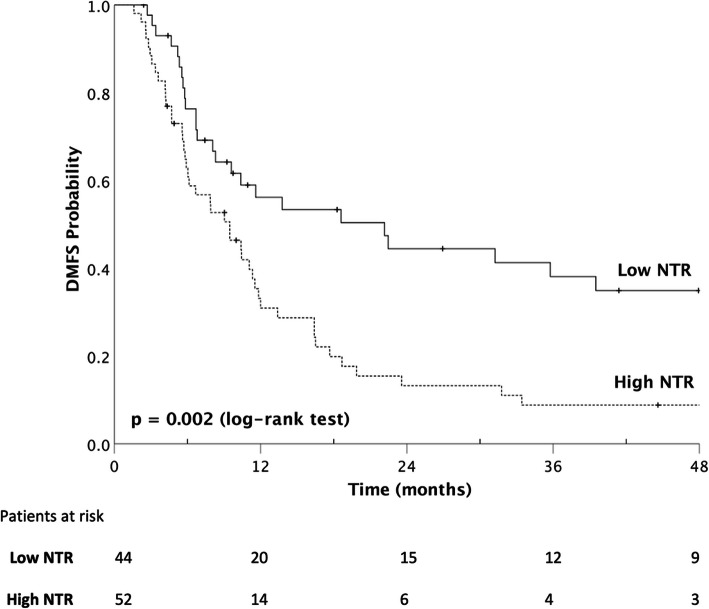
The Kaplan-Meier survival curves of the distant metastasis-free survival (median 9.5 vs. 22.2 months, *p* = 0.002 by log-rank test) of patients with unresectable esophageal cancer stratified according to node-to-tumor ratio of SUV (NTR) with cut-off value at 0.46

**Fig. 3:**
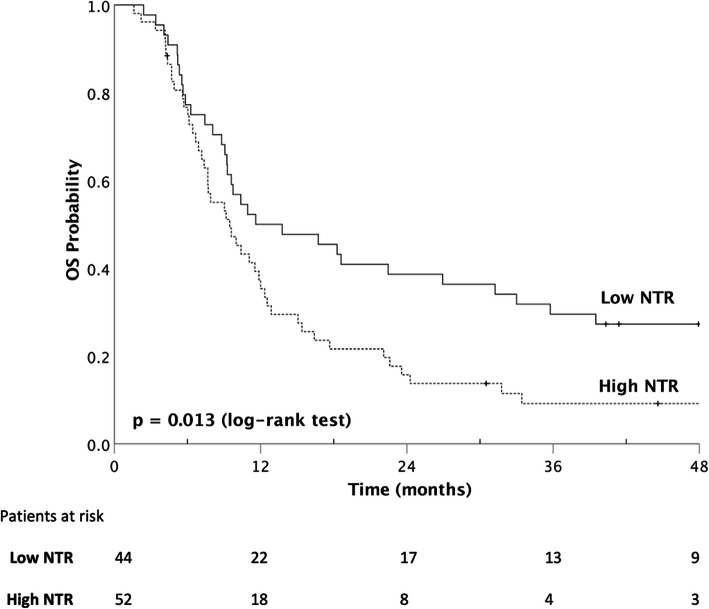
The Kaplan-Meier survival curves of the overall survival (median 9.5 vs. 11.6 months, *p* = 0.013 by log-rank test) of patients with unresectable esophageal cancer stratified according to node-to-tumor ratio of SUV (NTR) with cut-off value at 0.46

The median DMFS was significantly lower in the high-NTR group (9.5 vs. 22.2 months, *p* = 0.002, Fig. 2). Similar impacts of higher NTR could also be observed in OS (9.5 vs. 11.6 months, *p* = 0.013, Fig. 3), PFS (5.9 vs. 8.9 months, *p* = 0.030), and LRFFS (7.9 vs. 10.4 months, *p* = 0.017). The probabilities of 1-year and 2-year DMFS (high-NTR vs. low-NTR) were 30.9% vs. 56.2 and 13.3% vs. 44.5%. For OS, the probabilities were 35.4% vs. 50.0 and 15.7% vs. 38.6% for 1-year and 2-year, respectively.

Table 2 summarizes the results of univariable analysis of prognostic factors for DMFS and OS. In univariable analysis, dose less than 40 Gy in first course of radiotherapy (hazard ratio [HR] 1.72, *p* = 0.024), chemotherapy with cisplatin + 5-FU regimen (HR 2.30, *p* = 0.006), SUV_LN_ (HR 1.03, *p* = 0.026), and NTR (2.17, *p* = 0.002) were revealed as significantly negative prognostic factors for DMFS. Nodal stage, especially N3, was also found to have marginal impact (HR 1.86, *p* = 0.090) on DMFS. In aspect of OS, only NTR (HR 1.77, *p* = 0.014) was the significant prognostic variable while N3 disease (HR 2.06, *p* = 0.052), first course RT dose (HR 1.47, *p* = 0.091), chemotherapy regimen (HR 1.67, *p* = 0.053), and SUV_LN_ (HR 1.03, p = 0.053) were marginal.

**Table 2:** Univariable analysis of clinical variables with Cox proportional hazard model for DMFS and OS

Variables	DMFS		OS	
	HR (95% CI)	*P* value	HR (95% CI)	*P* value
**Gender (vs. Male)**				
Female	1.70 (0.77–3.71)	0.187	1.63 (0.75–3.55)	0.221
**Age (year)**	0.99 (0.96–1.02)	0.616	1.00 (0.97–1.03)	0.947
**Previous cancer history (vs. No)**				
Yes	0.59 (0.14–2.40)	0.456	0.53 (0.13–2.18)	0.383
**Performance status (vs. ECOG 0–1)**				
ECOG 2–3	1.54 (0.37–6.32)	0.552	2.43 (0.76–7.79)	0.135
**Differentiation (vs. Well to moderate)**				
Poorly differentiated	0.85 (0.47–1.51)	0.575	0.78 (0.45–1.36)	0.381
**Tumor length (cm)**	1.05 (0.94–1.16)	0.400	1.05 (0.95–1.16)	0.370
**Tumor location (vs. Cervical-upper)**				
Middle to lower third	0.71 (0.44–1.14)	0.158	0.82 (0.52–1.27)	0.366
**Nodal stage (vs. N0-N1)**				
N2	1.05 (0.52–2.12)	0.900	1.43 (0.72–2.86)	0.311
N3	1.86 (0.90–3.84)	0.090*	2.06 (0.99–4.28)	0.052*
**First course RT dose (vs. > = 40 Gy)**				
< 40 Gy	1.72 (1.08–2.75)	0.024**	1.47 (0.94–2.28)	0.091*
**Chemotherapy regimen (vs. Taxanes-based regimens)**				
Cisplatin + 5-FU	2.30 (1.27–4.17)	0.006**	1.67 (0.99–2.81)	0.053*
**SUV**_**Tumor**_	1.00 (0.97–1.04)	0.984	1.00 (0.96–1.03)	0.848
**SUV**_**LN**_	1.03 (1.00–1.07)	0.026**	1.03 (1.00–1.06)	0.053*
**NTR (vs. Low NTR [<= 0.46])**				
High NTR (> 0.46)	2.17 (1.33–3.53)	0.002**	1.77 (1.12–2.79)	0.014**

*DMFS* distant metastasis-free survival, *OS* overall survival, *HR* hazard ratio

* *p* value < 0.1 (marginal significance)

**p value < 0.05 (statistical significance)

In multivariable analysis, NTR (HR 1.81, *p* = 0.023) were found to have significant prognostic value in terms of DMFS. The usage of cisplatin + 5-FU regimen (HR 1.85, *P* = 0.052) conferred marginal significance, though. In the aspect of OS, NTR was the only independent prognostic parameter (HR 1.77, *p* = 0.014). The details of the multivariable analysis were listed in Table 3.

**Table 3:** Multivariable analysis of clinical variables with Cox proportional hazard model for DMFS and OS

Variables	DMFS		OS	
	HR (95% CI)	*P* value	HR (95% CI)	*P* value
**Nodal stage (vs. N0-N1)**		NS		NS
**First course RT dose (vs. > = 40 Gy)**		NS		NS
**Chemotherapy regimen (vs. Taxanes-based regimens)**		NS		NS
Cisplatin + 5-FU	1.85 (1.00–3.45)	0.052		NS
**SUV**_**LN**_		NS		NS
**NTR (vs. Low NTR [<= 0.46])**		NS		NS
High NTR (> 0.46)	1.81 (1.08–3.01)	0.023**	1.77 (1.12–2.79)	0.014**

*NS* the variable was removed during the multivariable analysis

**p value < 0.05 (statistical significance)

## Discussion

In this study, we aimed to investigate the prognostic value of pretreatment NTR in non-metastatic unresectable esophageal SCC. To our knowledge, it’s the first study focusing on PET parameters in patients with unresectable esophageal cancer. We demonstrated that the patients with NTR >  0.46 had a higher risk of developing distant metastasis and also a higher risk of mortality. Although patients with unresectable diseases were generally believed to have dismal outcomes, Cushman et al. [4] reported that patients receiving operation or definitive CRT have better survival outcomes compared to chemotherapy alone. The patient selection seemed to play a critical role. However, in our study, traditional risk factors like age, primary tumor size, and cell differentiation failed to show prognostic value, while nodal stage revealed only borderline significance. With mere traditional risk factors, the ability to predict patients’ outcomes is limited.

Different pretreatment PET parameters, e.g. SUV_LN_, metabolically active tumor volume (MTV), total lesion glycolysis (TLG), and standardized uptake ratio (SUR) were investigated for predicting esophageal cancer patient’s outcome as well. In the previous study conducted by Yap [16], the pretreatment SUV_LN_ rather than SUV_Tumor_ was found to correlate with the patient’s long-term outcome in terms of DMFS, PFS, and OS. Another study [17] found restaging MTV, TLG, SUV, and SUR were associated with better treatment outcomes, but concerning pretreatment parameters, only pretreatment MTV was prognostic for overall survival and locoregional control. Takahashi et al. [18] reported that only the sum of pretreatment MTV and TLG for all measurable lesions are independent predictors while SUV_Tumor_ is not. These findings are in line with other studies [19–21] revealing pretreatment SUV_Tumor_ has little prognostic value if used alone.

In our study, the DMFS (9.5 vs. 22.2 months, *p* = 0.002) and OS (9.5 vs. 11.6 months, *p* = 0.013) are significantly different when stratified by NTR. An important and fundamental question is that what is the additional benefits could be offered with NTR rather than SUV_LN_ alone. An aspect is the adaptation of NTR plays the role of internal control like other ratio methods do, which may reduce the factors that affecting SUV quantitation, e.g., tracer dose and patient’s weight uncertainties, acquisition time, and machine calibration [17]. Chung et al. conducted a study to evaluate the prognostic value of NTR in the endometrial cancer. In their report, NTR was found to have significant correlation with FIGO stage, LN metastasis, lymphovascular space invasion, recurrence, tumor grade, and deep myometrial invasion of tumor [14]. They proposed that relative metabolic activity could be s surrogate marker of tumor aggressiveness. Another possible and reasonable hypothesis is that NTR reflects the different behaviors between tumors with higher and lower metastatic potential. If the metastatic tumor of lymph nodes has higher relative metabolic activity as compared with the primary tumor, it may indicate higher inherited distant metastatic potential and may eventually lead to a poorer outcome. However, this hypothesis is not supported by solid evidence yet and further fundamental biological study is needed to prove this hypothesis.

Recently, utilizing induction chemotherapy first before aggressive local treatment has been proposed in patients with unresectable esophageal cancer. Makino et al. [22] showed that initial induction chemotherapy with docetaxel, cisplatin, and 5-fluorouracil (DCF) had better cancer-specific survival than CRT alone in patients with unresectable esophageal cancer. Yokota et al. [23] reported a multicenter phase 2 trial of induction DCF chemotherapy and subsequent conversion surgery in unresectable esophageal cancer, which showed a favorable survival outcome. For patients with high-NTR unresectable esophageal cancer, adequate systemic therapy is reasonably more important because of the high risk of distant metastases. Additionally, deferring upfront CRT may reduce unnecessary radiotherapy-related complications and toxicities to those who are doomed to distant failure. CRT-related complications for unresectable esophageal cancer, like aortic blow-out or tracheoesophageal fistula (TE fistula) [24], have always been a critical issue in the management of these patients. According to several studies [25–27] enrolling T4 esophageal cancer, the incidence of worsening or newly developed TE fistula during or after CRT was around 9–18 and 7% of treatment-related death was reported. We presume that the adoption of NTR would provide a better patient selection for more personalized treatment and may result in better outcomes of the patients with unresectable esophageal cancer.

Although we proposed a potential and easy-to-use parameter for unresectable esophageal SCC, there were several major limitations. The study was retrospectively designed and had unavoidable inherited selection bias. Second, we only focus on unresectable esophageal SCC in our study and whether the result is applicable to resectable disease is unknown. Third, all the patients were diagnosed and treated in one single medical center and the generalizability of the result may be limited. Further large-scale or prospective studies were needed to validate our findings.

In conclusion, we provided the first report of an easily accessible but potential PET parameter, NTR, for unresectable esophageal SCC. This group of patients has a dismal outcome and their optimal treatment has been controversial. Traditional risk factors provide limited prognostic information for this group of patients. The NTR could be an easy-to-use and helpful prognostic factor to decide optimal treatment for them.

## Conclusions

High pretreatment NTR (> 0.46) predicts worse treatment outcomes in terms of DMFS and OS in patients with non-metastatic and unresectable esophageal SCC. This group of patients has a dismal outcome and their optimal treatment has been controversial. NTR could be an easy-to-use and helpful prognostic factor to provide more personalized treatment for patients with unresectable esophageal SCC.

## Data Availability

The datasets used and/or analysed during the current study are available from the corresponding author on reasonable request.

## Abbreviations

5-FU: 5-fluorouracil
AUC: Area-under-curve
CI: Confidence interval
CRT: Chemoradiotherapy
DMFS: Distant metastasis-free survival
FDG PET/CT: 2-deoxy-2-[18F]fluoro-D-glucose positron emission tomography/computed tomography
HR: Hazard ratio
IQR: Interquartile range
LRFFS: Local regional failure-free survival
MTV: Metabolic tumor volume
NTR: Node-to-tumor SUV ratio
OS: Overall survival
OSEM: Ordered-subset expectation maximization
PFS: Progression-free survival
ROC: Analysis receiver operating characteristic analysis
SCC: Squamous cell carcinoma
SUR: Standardized uptake ratio
SUV: Maximum standardized uptake value
SUV_LN_: Maximum standardized uptake value of metastatic lymph nodes
SUV_Tumor_: Maximum standardized uptake value of primary tumor
TE fistula: Tracheoesophageal fistula
TLG: Total lesion glycolysis

## References

[1] Bray F, Ferlay J, Soerjomataram I, Siegel RL, Torre LA, Jemal A (2018). Global cancer statistics 2018: GLOBOCAN estimates of incidence and mortality worldwide for 36 cancers in 185 countries. CA Cancer J Clin.

[2] Arnold M, Soerjomataram I, Ferlay J, Forman D (2015). Global incidence of oesophageal cancer by histological subtype in 2012. Gut..

[3] Wu SG, Zhang WW, Sun JY, Li FY, Lin Q, He ZY (2018). Patterns of Distant Metastasis Between Histological Types in Esophageal. Cancer Front Oncol.

[4] Cushman TR, Shaaban SG, Moreno AC, Lin C, Verma V (2019). Management of Unresectable T4b esophageal Cancer: practice patterns and outcomes from the National Cancer Data Base. Am J Clin Oncol.

[5] Yamaguchi S, Morita M, Yamamoto M, Egashira A, Kawano H, Kinjo N (2018). Long-term outcome of definitive Chemoradiotherapy and induction Chemoradiotherapy followed by surgery for T4 esophageal Cancer with tracheobronchial invasion. Ann Surg Oncol.

[6] Meyers BF, Downey RJ, Decker PA, Keenan RJ, Siegel BA, Cerfolio RJ (2007). The utility of positron emission tomography in staging of potentially operable carcinoma of the thoracic esophagus: results of the American College of Surgeons oncology group Z0060 trial. J Thorac Cardiovasc Surg.

[7] Lordick F, Ott K, Krause B-J, Weber WA, Becker K, Stein HJ (2007). PET to assess early metabolic response and to guide treatment of adenocarcinoma of the oesophagogastric junction: the MUNICON phase II trial. Lancet Oncol.

[8] Yap WK, Chang YC, Hsieh CH, Chao YK, Chen CC, Shih MC (2018). Favorable versus unfavorable prognostic groups by post-chemoradiation FDG-PET imaging in node-positive esophageal squamous cell carcinoma patients treated with definitive chemoradiotherapy. Eur J Nucl Med Mol Imaging.

[9] Greally M, Ku GY (2018). Metabolic assessment by PET in the treatment of esophageal cancer. Ann Esophagus.

[10] Schollaert P, Crott R, Bertrand C, D'Hondt L, Borght TV, Krug B (2014). A systematic review of the predictive value of (18) FDG-PET in esophageal and esophagogastric junction cancer after neoadjuvant chemoradiation on the survival outcome stratification. J Gastrointest Surg.

[11] Futamura M, Asano T, Kobayashi K, Morimitsu K, Nawa M, Kanematsu M (2015). Prediction of macrometastasis in axillary lymph nodes of patients with invasive breast cancer and the utility of the SUV lymph node/tumor ratio using FDG-PET/CT. World J Surg Oncol.

[12] Kim YH, Yoon HJ, Kim Y, Kim BS (2015). Axillary lymph node-to-primary tumor standard uptake value ratio on preoperative (18) F-FDG PET/CT: a prognostic factor for invasive ductal breast Cancer. J Breast Cancer.

[13] Cho J, Choe JG, Pahk K, Choi S, Kwon HR, Eo JS (2017). Ratio of Mediastinal lymph node SUV to primary tumor SUV in (18) F-FDG PET/CT for nodal staging in non-small-cell lung Cancer. Nucl Med Mol Imaging.

[14] Chung HH, Cheon GJ, Kim JW, Park NH, Song YS (2018). Prognostic value of lymph node-to-primary tumor standardized uptake value ratio in endometrioid endometrial carcinoma. Eur J Nucl Med Mol Imaging.

[15] Chung HH, Cheon GJ, Kim JW, Park NH, Song YS (2017). Prognostic importance of lymph node-to-primary tumor standardized uptake value ratio in invasive squamous cell carcinoma of uterine cervix. Eur J Nucl Med Mol Imaging.

[16] Yap WK, Chang YC, Tseng CK, Hsieh CH, Chao YK, Su PJ (2017). Predictive value of nodal maximum standardized uptake value of pretreatment [18F] fluorodeoxyglucose positron emission tomography imaging in patients with esophageal cancer. Dis Esophagus.

[17] Butof R, Hofheinz F, Zophel K, Schmollack J, Jentsch C, Zschaeck S, et al. Prognostic value of SUR in patients with trimodality treatment of locally advanced esophageal carcinoma. J Nucl Med. 2019;60(2):192–8. 10.2967/jnumed.117.207670 PMC8833854 30166358

[18] Takahashi N, Umezawa R, Takanami K, Yamamoto T, Ishikawa Y, Kozumi M (2018). Whole-body total lesion glycolysis is an independent predictor in patients with esophageal cancer treated with definitive chemoradiotherapy. Radiother Oncol.

[19] Mamede M, Abreu ELP, Oliva MR, Nose V, Mamon H, Gerbaudo VH (2007). FDG-PET/CT tumor segmentation-derived indices of metabolic activity to assess response to neoadjuvant therapy and progression-free survival in esophageal cancer: correlation with histopathology results. Am J Clin Oncol.

[20] Arnett ALH, Merrell KW, Macintosh EM, James SE, Nathan MA, Shen KR (2017). Utility of (18) F-FDG PET for predicting Histopathologic response in esophageal carcinoma following Chemoradiation. J Thorac Oncol.

[21] Schmidt T, Lordick F, Herrmann K, Ott K (2015). Value of functional imaging by PET in esophageal Cancer. J Natl Compr Cancer Netw.

[22] Makino T, Yamasaki M, Miyazaki Y, Wada N, Takahashi T, Kurokawa Y, et al. Utility of initial induction chemotherapy with 5-fluorouracil, cisplatin, and docetaxel (DCF) for T4 esophageal cancer: a propensity score-matched analysis. Dis Esophagus. 2017;31(4):1–7. 10.1093/dote/dox130 29190316

[23] Yokota T, Kato K, Hamamoto Y, Tsubosa Y, Ogawa H, Ito Y, et al. A 3-year overall survival update from a phase 2 study of Chemoselection with DCF and subsequent conversion surgery for locally advanced Unresectable esophageal cancer. Ann Surg Oncol. 2020;27(2):460–7. 10.1245/s10434-019-07654-8 31376034

[24] Ku GY, Goodman KA, Rusch VW, Ilson DH (2009). Successful treatment of esophageal Cancer with airway invasion with induction chemotherapy and concurrent Chemoradiotherapy. J Thorac Oncol.

[25] Kaneko K, Ito H, Konishi K, Kurahashi T, Ito T, Katagiri A (2003). Definitive chemoradiotherapy for patients with malignant stricture due to T3 or T4 squamous cell carcinoma of the oesophagus. Br J Cancer.

[26] Ohtsu A, Boku N, Muro K, Chin K, Muto M, Yoshida S (1999). Definitive Chemoradiotherapy for T4 and/or M1 Lymph Node Squamous Cell Carcinoma of the Esophagus. J Clin Oncol.

[27] Nishimura Y, Suzuki M, Nakamatsu K, Kanamori S, Yagyu Y, Shigeoka H (2002). Prospective trial of concurrent chemoradiotherapy with protracted infusion of 5-fluorouracil and cisplatin for T4 esophageal cancer with or without fistula. Int J Radiat Oncol Biol Phys.

